# Daddy is dead: a physician reckons with her father’s mortality

**DOI:** 10.1093/oncolo/oyag210

**Published:** 2026-05-29

**Authors:** Oyepeju Abioye

**Affiliations:** Department of Internal Medicine, Allegheny Health Network, Pittsburgh, PA 15212, United States

Some memories burn themselves so deeply into your consciousness that time cannot fade them. For me, it was 9:09 am on a Thursday, a moment forever crystallized in my mind. The night before, I learned my father’s hemoglobin had dropped to 4%. As a newly minted physician, I knew what that number meant. As a daughter, I prayed I was wrong. I had thrown clothes into a duffle bag and begun the journey home, hoping I would make it in time.

In the taxi the next morning, music from the stereo faded to background noise as I answered my phone. My mother’s voice, broken by sobs, changed everything: *“Daddy is dead.”* The scream that erupted from me was primal, raw; a sound that still echoes in my dreams. Even now, years later, I can feel the exact texture of that moment: the cool leather of the taxi seat, the way my fingers clutched my hastily packed bag, the terrible clarity of knowing I was too late. The four-hour journey home became an endless corridor of memories. Each mile marked another moment I would never share with him, another chance lost to hold his hand one last time. His final *“I love you”* played on repeat in my mind. It was a blessing that would become both treasure and torment, a last gift clutched close while mourning all the *“I love you’s”* that would never come.

Years later, walking across the hospital’s sterile processing unit, the word *“sterile”* jumps out at me. It reminds me of something I consciously decided not to be: a clear-cut, mechanical person. I don’t remember with precision when I made that choice, but I know it had everything to do with my father’s death. I think of him often in these clinical spaces, where life and death intertwine in measured numbers and calculated risks.

The first poem I ever wrote was about my father. It wasn’t a very good poem, but even then, as a seven-year-old, I remember giving it to him and watching his face break into the widest grin possible. This memory stands in stark contrast to another: watching him experience what I now categorize as pre-death, that awareness when death approaches and you know it. I picture him wistfully staring into the distance, scared to his now-weakened bones from prostate cancer metastasis, unable to fathom the why, the how, or the when.

These fragments of memory: a childhood poem, a clinical number, a man facing his mortality; have shaped not just who I am as a physician but how I approach the human beings behind the diagnoses. Few, if any, medical texts capture what it feels like to watch your father fade while your professional knowledge narrates every step of his decline. They don’t tell you how to handle the guilt of being both a physician and a helpless child. They don’t prepare you for the way grief and medicine intertwine, how every critical lab value becomes personal, how every terminal diagnosis reminds you of your own loss.

The tragedy of my father’s late diagnosis reflects a broader crisis in African healthcare. When routine cancer screening is viewed as a luxury rather than a necessity, early detection becomes a privilege few can access, and symptoms often go unrecognized until the disease has advanced significantly. Like many African men of his generation, my father had never undergone prostate cancer screening. By the time he sought medical help, Prostate Specific Antigen (PSA) results exceeded 1000, a number that still haunts my medical consciousness, knowing the normal range is 0-4. When faced with treatment options for castrate-resistant prostate cancer, his choice challenged my medical training but reflected deep-seated cultural realities. After experiencing some side effects of conventional medical treatment options, he opted for Complementary and Alternative Medicine (CAM). This decision, while difficult to accept, made sense within the context of a profound mistrust of Western medical interventions, rooted in historical experiences.

I wrestled with adequately capturing this intersection of personal autonomy, cultural context, medical infrastructure deficits, and terminal illness. How do we bridge the gap between evidence-based medicine and deeply held cultural beliefs about healing? These questions become even more gut-wrenching when the patient is your father, and you must balance your roles as both a physician and a child. Alas, I finally understood that medicine often bows at the feet of cultural competency and patient choice.

Medicine, I’ve learned, lives in the space between science and story. My father’s journey taught me that being a physician means more than understanding pathology and treatment protocols. It means recognizing that each patient carries their own constellation of beliefs, fears, and hopes shaped by culture, circumstance, and personal truth. Now, years later, when I’m involved in counseling patients about cancer screening or discussing treatment options with them, I carry my father’s story like a compass. It guides me through difficult conversations, helping me navigate the delicate balance between medical knowledge and human understanding. I see him in the faces of fathers who sit across from me in the clinic, in children who advocate for their parents, and in families grappling with decisions no textbook can simplify. This is my father’s immortal gift to me: the understanding that behind every medical chart lies a human story, waiting to be heard, deserving to be honored.

